# Increased monocyte count and red cell distribution width as prognostic biomarkers in patients with Idiopathic Pulmonary Fibrosis

**DOI:** 10.1186/s12931-021-01725-9

**Published:** 2021-05-05

**Authors:** Theodoros Karampitsakos, Sebastiano Torrisi, Katerina Antoniou, Effrosyni Manali, Ioanna Korbila, Ourania Papaioannou, Fotios Sampsonas, Matthaios Katsaras, Eirini Vasarmidi, Despoina Papakosta, Kalliopi Domvri, Eva Fouka, Ioannis Organtzis, Zoe Daniil, Ilias Dimeas, Paraskevi Kirgou, Konstantinos I. Gourgoulianis, Ilias C. Papanikolaou, Katerina Markopoulou, Georgia Kounti, Eirini Tsapakidou, Efthymia Papadopoulou, Konstantinos Tatsis, Athena Gogali, Konstantinos Kostikas, Vasilios Tzilas, Serafeim Chrysikos, Spyridon Papiris, Demosthenes Bouros, Michael Kreuter, Argyrios Tzouvelekis

**Affiliations:** 1 Department of Respiratory Medicine, University Hospital of Patras, Patras, Greece; 2 Center for Interstitial and Rare Lung Diseases, Pneumology, Thoraxklinik, University of Heidelberg, Heidelberg, Germany; 3 Laboratory of Molecular and Cellular Pneumonology, Department of Respiratory Medicine, Faculty of Medicine, University of Crete, Heraklion, Crete Greece; 4 2nd Pulmonary Medicine Department, “ATTIKON” University Hospital, Athens Medical School, National and Kapodistrian University of Athens, Athens, Greece; 5 Pulmonary Department, Medical School, Aristotle University of Thessaloniki, “G. PAPANIKOLAOU’’ General Hospital, Exochi, Thessaloniki, Greece; 6 Department of Respiratory Medicine, Medical School, University of Thessaly, Larissa, Greece; 7 Respiratory Medicine Department, “Corfu General Hospital”, Corfu, Greece; 8 Pulmonary Department “G. PAPANIKOLAOU” General Hospital, Thessaloniki, Greece; 9 Department of Respiratory Medicine, Medical School, University of Ioannina, Ioannina, Greece; 10 First Academic Department of Pneumonology, Hospital for Thoracic Diseases, “SOTIRIA”, Medical School, National and Kapodistrian University of Athens, Athens, Greece; 11 5th Department of Pneumonology, Hospital for Thoracic Diseases, “SOTIRIA”, Athens, Greece; 12 German Center for Lung Research, Heidelberg, Germany

**Keywords:** Monocyte count, RDW, Idiopathic pulmonary fibrosis, Biomarkers, Mortality

## Abstract

**Background:**

Idiopathic Pulmonary Fibrosis (IPF) represents a chronic lung disease with unpredictable course.

**Methods:**

We aimed to investigate prognostic performance of complete blood count parameters in IPF. Treatment-naïve patients with IPF were retrospectively enrolled from two independent cohorts (derivation and validation) and split into subgroups (high and low) based on median baseline monocyte count and red cell distribution width (RDW).

**Results:**

Overall, 489 patients (derivation cohort: 300, validation cohort: 189) were analyzed. In the derivation cohort, patients with monocyte count ≥ 0.60 K/μL had significantly lower median FVC%pred [75.0, (95% CI 71.3–76.7) vs. 80.9, (95% CI 77.5–83.1), (*P* = 0.01)] and DLCO%pred [47.5, (95% CI 44.3–52.3) vs. 53.0, (95% CI 48.0–56.7), (*P* = 0.02)] than patients with monocyte count < 0.60 K/μL. Patients with RDW ≥ 14.1% had significantly lower median FVC%pred [75.5, (95% CI 71.2–79.2) vs. 78.3, (95% CI 76.0–81.0), (*P* = 0.04)] and DLCO%pred [45.4, (95% CI 43.3–50.5) vs. 53.0, (95% CI 50.8–56.8), (*P* = 0.008)] than patients with RDW < 14.1%. Cut-off thresholds from the derivation cohort were applied to the validation cohort with similar discriminatory value, as indicated by significant differences in median DLCO%pred between patients with high vs. low monocyte count [37.8, (95% CI 35.5–41.1) vs. 45.5, (95% CI 41.9–49.4), (*P* < 0.001)] and RDW [37.9, (95% CI 33.4–40.7) vs. 44.4, (95% CI 41.5–48.9), (*P* < 0.001)]. Patients with high monocyte count and RDW of the validation cohort exhibited a trend towards lower median FVC%pred (*P* = 0.09) and significantly lower median FVC%pred (*P* = 0.001), respectively. Kaplan–Meier analysis in the derivation cohort demonstrated higher all-cause mortality in patients with high (≥ 0.60 K/μL) vs. low monocyte count (< 0.60 K/μL) [HR 2.05, (95% CI 1.19–3.53), (*P* = 0.01)].

**Conclusions:**

Increased monocyte count and RDW may represent negative prognostic biomarkers in patients with IPF.

**Supplementary Information:**

The online version contains supplementary material available at 10.1186/s12931-021-01725-9.

## Background

Idiopathic pulmonary fibrosis (IPF) represents a chronic, progressive lung disease with dismal prognosis despite the advent of novel antifibrotic compounds [1–4]. The disease course is highly variable and prognosis remains challenging [5]. A clinicians’ friendly, easily applicable and cost-effective prognostic biomarker with uniform cut-off values that will guide disease stratification and tailoring of therapeutic approaches is missing [6]. Several biomarkers including Mucin 5b and Toll-interacting protein single nucleotide polymorphisms, telomere length and gene expression signatures have been suggested as reliable prognosticators in patients with IPF [7–18]; yet, measurement of such biomarkers remains laborious and requires expensive and sophisticated infrastructure. In addition, lack of standardization of samples’ collection protocols leading to non-reproducible cut-off thresholds, further limits their widespread clinical applicability [6, 19].

Abundant evidence has highlighted the role of Complete Blood Count (CBC) in the prognostication of patients with various chronic lung diseases [6, 20, 21]. Asthma researchers are currently using peripheral eosinophils to implement anti-IL5/13 therapeutic regimens [22]. Three major studies, encompassing an overall of almost 10,000 patients with IPF and scleroderma-associated interstitial lung disease have recently identified elevated peripheral blood monocyte count as a biomarker of disease progression and mortality [6, 23]. Given that monocyte count is a clinically applicable and inexpensive biomarker, these findings warrant further investigation in real-life studies. In line with this concept another parameter of CBC, red cell distribution width (RDW), has been associated with worse clinical outcomes in several chronic lung diseases including IPF and chronic obstructive pulmonary disease [20, 24–26]. Increased RDW seems to represent a biomarker of early hypoxemia [20, 27, 28].

To this end, our aim was to evaluate the prognostic role of parameters of CBC, including monocyte count and RDW in two independent cohorts (derivation and validation) of patients with IPF in a real-life clinical setting.

## Study design and methods

This was an observational, retrospective study. Between 01/11/2018 and 31/08/2020, we retrospectively enrolled patients with IPF and available CBC at baseline (prior to anti-fibrotic treatment), as well as 6 and 12 months post-treatment. Only treatment-naïve patients at the time of baseline CBC were included in the analysis. There were no patients receiving antifibrotics or corticosteroids at the time of baseline CBC. Epidemiological data were derived from two independent cohorts.

*Derivation cohort* The derivation cohort included patients from referral centers for Interstitial Lung Diseases in Greece including Department of Internal and Respiratory Medicine, University Hospital of Patras, 1st and 2nd Academic Department of Respiratory Medicine, “SOTIRIA” and “ATTIKON” General Hospital, National and Kapodistrian University of Athens, Laboratory of Molecular and Cellular Pneumonology, Department of Respiratory Medicine, Faculty of Medicine, University of Crete, Heraklion, Crete, Medical School, University of Thessaly, Larissa, Respiratory Medicine Department, “Corfu General Hospital”, Department of Respiratory Medicine, “G. PAPANIKOLAOU” General Hospital, Thessaloniki, Aristotle University of Thessaloniki and Department of Respiratory Medicine, Medical School, University of Ioannina.

*Validation cohort* The validation cohort included patients from the Center for interstitial and rare lung diseases, Pneumology, Thoraxklinik, University of Heidelberg, Germany and German Center for Lung Research, Heidelberg, Germany.

The study was approved by the Institutional Review Board and the Local Ethics Committee (Protocol Number: 458/06-12-19). Diagnosis of IPF was based on ATS/ERS/JRS/ALAT guidelines [1]. We collected parameters of CBC including monocyte count and RDW prior anti-fibrotic treatment, as well as 6 and 12 months post-treatment. Baseline demographics and comorbid conditions were recorded. Pulmonary hypertension was defined as elevated right ventricular systolic pressure on echocardiographic assessment, as all patients performed a baseline echocardiography but not right heart catheterization.

### Statistical analysis

Median values of CBC parameters were recorded. Median values were used, as Kolmogorov–Smirnov test for normal distribution rejected normality. Patients were divided in subgroups based on the median value of each CBC parameter in the derivation cohort (high and low). We used median values based on the fact that monocyte count and RDW did not have significant differences over the 1-year period both in our cohorts and in other studies [25, 29]. Mann–Whitney test was applied to assess differences in Forced Vital Capacity %predicted (FVC%pred) and Diffusion capacity of lung for carbon monoxide %predicted (DLCO%pred) between subgroups of patients split by the median value of CBC parameters. Prognostic performance of these cut-off thresholds was also assessed in the validation cohort. Kaplan–Meier survival analysis was applied to investigate differences in survival probability between high and low subgroups. Kaplan–Meier was also used to dichotomize patients based on the previously published cut-off threshold of monocyte count (0.95 K/μL) [6]. Differences in parameters of CBC between patients in need of Long Term Oxygen Therapy (LTOT) and patients without LTOT were investigated with Mann–Whitney. *P* -values < 0.05 were considered statistically significant.

## Results

### Patient baseline characteristics

Overall, 489 patients (derivation cohort: N = 300, validation cohort: N = 189) were included in the analysis. Patient demographics and disease characteristics are summarized in Table 1. Median age (95% CI) was 74 (73–75) years for the derivation cohort and 74 (72–75) years for the validation cohort. Patients with IPF were predominantly male both in the derivation (83.3%, N = 250) and validation cohort (78.8%, N = 149). Median monocyte count (95% CI) was 0.60 (0.57–0.62) and 0.52 (0.50–0.58) K/μL for the derivation and the validation cohort, respectively. Median RDW (95% CI) for the derivation cohort was 14.1% (13.9–14.3) and 13.7% (13.6–13.8) for the validation cohort. Finally, median FVC%pred (95% CI) was 77.0 (75.0–79.8) and 76.2 (71.7–80.8), while median DLCO%pred (95% CI) was 51.0 (47.1–53.8) and 41.9 (40.3–44.9) for the derivation and validation cohort, respectively (Table 1). Median follow-up (95% CI) was 24.3 (23.4–28.7) and 15.0 (12.0–19.0) months for the derivation and the validation cohort, respectively.

**Table 1:** Baseline characteristics of patients enrolled in the study

Characteristics	Derivation cohort (N, %)	Validation cohort (N, %)	*P* value
Number of patients	300	189	NA
Median age (%95 CI)	74 (73 to 75)	74 (72–75)	0.14
Males/Females	250 (83.3)/50 (16.7)	149 (78.8)/40 (22.2)	NA
Current	38 (12.7)	1 (0.5)	NA
Ex-smokers	199 (66.3)	131 (69.3)	NA
Never smokers	63 (21.0)	57 (30.2)	NA
Median monocyte count (K/μL) (95% CI)	0.60 (0.57–0.62)	0.52 (0.50–0.58)	**0.002**
Median RDW (95% CI)	14.1 (13.9–14.3)	13.7 (13.6–13.8)	** < 0.001**
Median FVC%pred (95% CI)	77.0 (75.0–79.8)	76.2 (71.7–80.8)	0.69
Median DLCO%pred (95% CI)	51.0 (47.1–53.8)	41.9 ( 40.3–44.9)	** < 0.001**
Arterial Hypertension	171 (57.0)	111 (58.7)	NA
Pulmonary Hypertension	55 (18.3)	9 (4.8)	NA
GERD	89 (29.6)	26 (13.8)	NA
Diabetes Mellitus	65 (21.7)	51 (27.0)	NA
Thyroid Disorders	25 (8.3)	22 (11.6)	NA
LTOT	49 (16.3)	65 (43.4)	NA
Nintedanib	123 (41.0)	76 (40.2)	NA
Pirfenidone	147 (49.0)	90 (47.6)	NA

Statistically significant *P*-values are shown in bold

*CI* confidence interval, *DLCO* diffusing capacity for carbon monoxide, *FVC* forced vital capacity, *GERD* gastroesophageal reflux disease, *LTOT* long term oxygen therapy, *RDW* red cell distribution width

### Functional indices at baseline

#### Patients in the high monocyte and RDW group exhibited more advanced disease at baseline

**Fig. 1:**
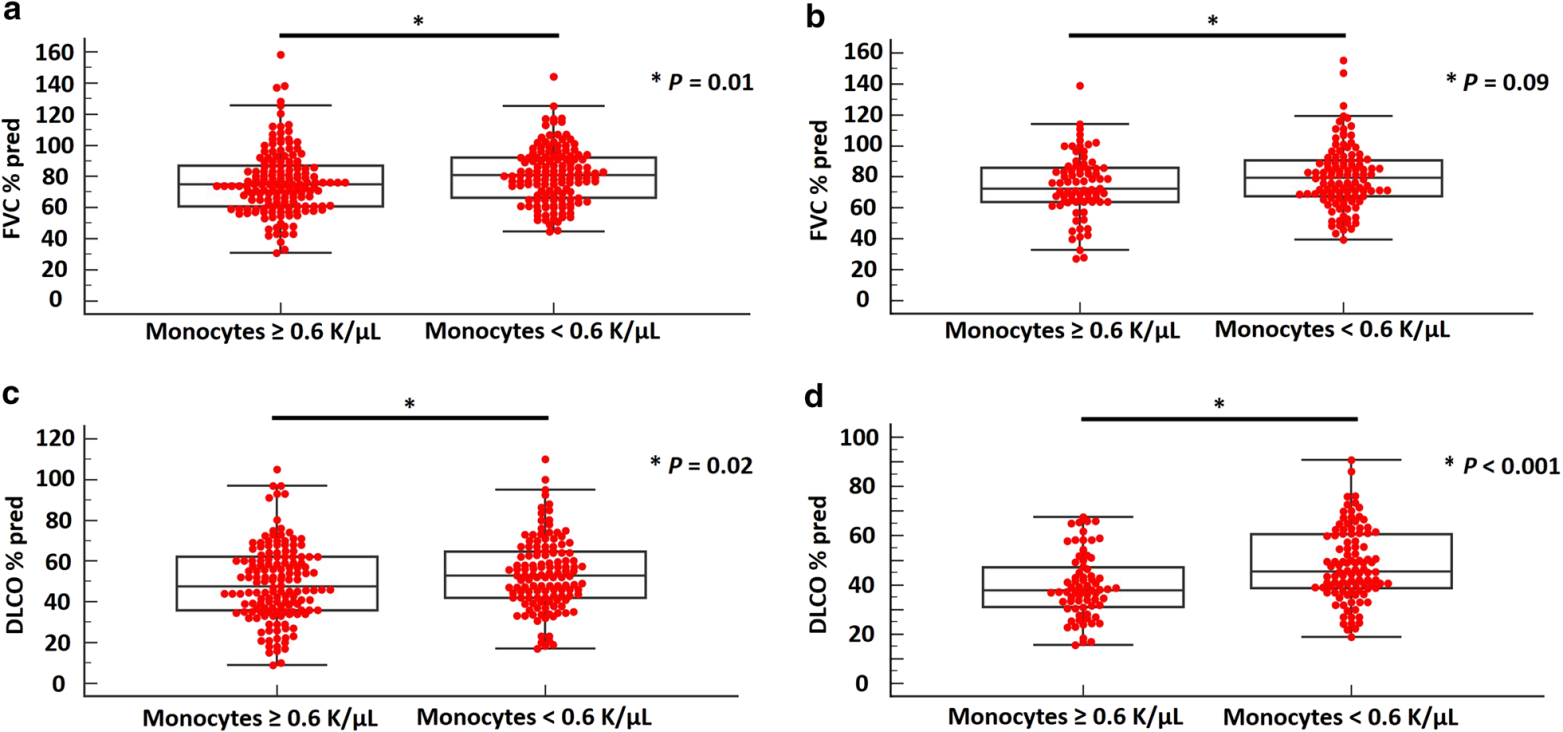
In the derivation cohort, patients with monocyte count ≥ 0.60 K/μL presented with significantly lower median FVC%pred than patients with monocyte count < 0.60 K/μL [75.0, (95% CI 71.3–76.7) vs. 80.9, (95% CI 77.5–83.1), (*P* = 0.01)] (**a**). In the validation cohort, median FVC%pred was 72.4 (95% CI 68.8 to 79.6) and 79.5 (95% CI 72.9–82.9) for patients with baseline monocyte count ≥ 0.60 K/μL and < 0.60 K/μL, respectively, (*P* = 0.09), (**b**). In the derivation cohort, patients with monocyte count ≥ 0.60 K/μL presented with significantly lower median DLCO%pred than patients with monocyte count < 0.60 K/μL [47.5, (95% CI 44.3–52.3) vs. 53.0, (95% CI 48.0–56.7), (*P* = 0.02)] (**c**). The same cut-off threshold had similar discriminatory value in the validation cohort [median DLCO%pred for patients with baseline monocyte count ≥ 0.60 K/μL: 37.8, (95% CI 35.5 to 41.1) vs. median DLCO%pred for patients with baseline monocyte count < 0.60 K/μL: 45.5, (95% CI 41.9 to 49.4), (*P* < 0.001), (**d**)]

In the derivation cohort, patients in the high monocyte group (≥ 0.60 K/μL) presented with significantly lower median FVC%pred [75.0, (95% CI 71.3–76.7)] and DLCO%pred [47.5, (95% CI 44.3–52.3)] compared to patients in the low monocyte group (< 0.60 K/μL) [80.9, (95% CI 77.5–83.1), *P* = 0.01 and 53.0 (95% CI 48.0–56.7), *P* = 0.02, respectively], (Fig. 1a, c). In the validation cohort, a trend towards lower FVC%pred [median: 72.4, (95% CI 68.8–79.6)] in the high monocyte group (≥ 0.60 K/μL) compared to the low monocyte group [median: 79.5, (95% CI 72.9–82.9)] was observed; yet, no statistical significance was reached (*P* = 0.09), (Fig. 1b). Importantly, patients in the high monocyte group of the validation cohort, exhibited significantly lower baseline median DLCO%pred compared to patients in the low monocyte group [37.8, (95% CI 35.5–41.1) vs. 45.5, (95% CI 41.9–49.4), (*P* < 0.001), (Fig. 1d)].

**Fig. 2:**
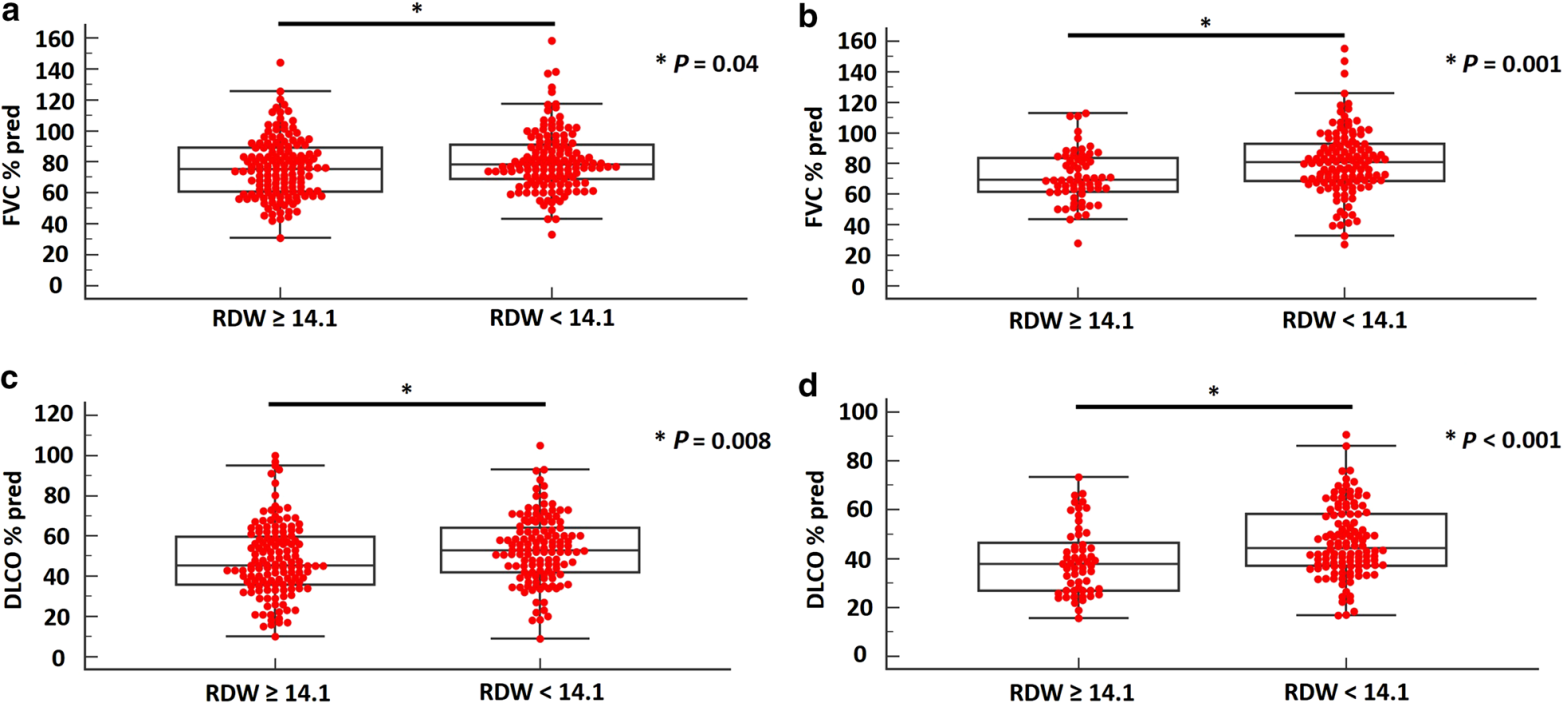
In the derivation cohort, patients with RDW ≥ 14.1% had significantly lower median FVC%pred than patients with RDW < 14.1% [75.5, (95% CI 71.2–79.2) vs. 78.3, (95% CI 76.0–81.0), (*P* = 0.04)] (**a**). Patients with baseline RDW ≥ 14.1% had significantly lower median FVC%pred [69.4, (95% CI 65.5–76.4)] compared to patients with baseline RDW < 14.1% [80.8, (95% CI 76.0–83.3)] in the validation cohort, (*P* = 0.001), (**b**). In the derivation cohort, patients with RDW ≥ 14.1% had significantly lower median DLCO%pred than patients with RDW < 14.1% [45.4, (95% CI 43.3–50.5) vs. 53.0, (95% CI 50.8–56.8), (*P* = 0.008)] (**c**). Median DLCO%pred was also lower for patients with baseline RDW ≥ 14.1% [37.9, (95% CI 33.4–40.7)] compared to patients with baseline RDW < 14.1% [44.4, (95% CI 41.5–48.9)] in the validation cohort, (*P* < 0.001), (**d**)

Similarly to what has been reported for monocyte count, patients in the high RDW group (≥ 14.1%) exhibited significantly lower baseline median FVC%pred [75.5, (95% CI 71.2–79.2) vs. 78.3, (95% CI 76.0–81.0), (*P* = 0.04), and 69.4, (95% CI 65.5–76.4) vs. 80.8, (95% CI 76.0–83.3), *P* = 0.001] and DLCO%pred, [45.4, (95% CI 43.3–50.5) vs. 53.0, (95% CI 50.8–56.8), (*P* = 0.008) and 37.9, (95% CI 33.4–40.7) vs. 44.4, (95% CI 41.5–48.9), (*P* < 0.001)], in both the derivation and the validation cohort, respectively (Fig. 2a–d).

Multiple regression analysis of the overall study population showed that baseline monocyte count was independently associated with baseline DLCO%pred (*P* = 0.005). With regards to other factors, gender was independently associated with baseline FVC%pred (*P* = 0.007), while presence of pulmonary hypertension was independently associated with baseline DLCO%pred (*P* = 0.002) (Table 2). Moreover, multiple regression analysis investigating the impact of comorbidities on baseline CBC values, showed that presence of pulmonary hypertension was independently associated with baseline RDW (*P* < 0.001) (Additional file 1: Table S1).

**Table 2:** Multiple regression analysis of studied biomarkers adjusted for confounding factors in the overall population

Parameter	FVC%pred			DLCO%pred		
	Coefficient	Std Error	p value	Coefficient	Std Error	*P* value
Monocytes	− 3.5296	4.8363	0.47	− 12.0046	4.2103	**0.005**
RDW	− 1.1988	0.6512	0.07	− 0.8736	0.5685	0.13
Hb	0.1269	0.5416	0.81	− 0.4136	0.4689	0.38
Age	− 0.08382	0.1283	0.51	− 0.1272	0.1109	0.25
Gender	9.2972	2.7196	**0.0007**	− 2.356	2.4101	0.33
Current smoker	19.3400	10.6770	0.07	− 11.2809	9.0903	0.22
Ever smoker	9.4636	9.8694	0.34	− 13.1024	8.4037	0.12
Never smoker	6.3952	10.0335	0.52	− 13.143	8.5571	0.13
Prior steroid use	− 8.6854	5.9834	0.15	− 0.02734	5.1068	0.99
AH	− 0.9292	2.1128	0.66	2.8003	1.8596	0.13
PH	− 3.9779	2.9795	0.18	− 8.2346	2.6026	**0.002**
GERD	0.5718	2.3002	0.80	2.5991	2.0101	0.20
DM	− 3.2035	2.4011	0.18	− 3.7688	2.1474	0.08
Thyroid disorders	0.6137	3.4471	0.86	1.7218	2.9847	0.56

Statistically significant *P*-values are shown in bold

*AH* arterial hypertension, *DLCO* diffusing capacity for carbon monoxide, *DM* diabetes mellitus, *FVC* forced vital capacity, *GERD* gastroesophageal reflux disease, *Hb* hemoglobin, *PH* pulmonary hypertension, *RDW* red cell distribution width, *Std* standard

Median monocyte count (K/μL) was significantly higher for patients in need of LTOT compared to those not in need of LTOT both in the derivation [0.70, (95% CI 0.64–0.80) vs. 0.56, (95% CI 0.53–0.60), (*P* < 0.001)] and validation cohort [0.60, (95% CI 0.54–0.60) vs. 0.50, (95% CI 0.44–0.52), (*P* = 0.004)]. Median RDW% was also significantly higher for patients in need of LTOT compared to those not receiving LTOT [derivation cohort: 15.1, (95% CI 14.2–15.4) vs. 13.9, (95% CI 13.7–14.1), (*P* = 0.002)], [validation cohort: 13.8, (95% CI 13.6–14.3) vs. 13.5 (95% CI 13.2–13.8), (*P* = 0.003)], (Table 3).

**Table 3:** Monocyte count and RDW in subgroup of patients based on the need of LTOT

	LTOT	NO LTOT	*P* value
Median Monocyte count (derivation cohort), (95% CI)	0.70 (0.64–0.80)	0.56 (0.53–0.60)	< 0.001
Median Monocyte count (validation cohort), (95% CI)	0.60 (0.54–0.60)	0.50 (0.44–0.52)	0.004
Median RDW (derivation cohort), (95% CI)	15.1 (14.2–15.4)	13.9 (13.7–14.1)	0.002
Median RDW (validation cohort), (95% CI)	13.8 (13.6–14.3)	13.5 (13.2–13.8)	0.003

*CI* confidence interval, *LTOT* long term oxygen therapy, *RDW* red cell distribution width

### Disease progression

#### Monocyte count and RDW were not associated with disease progression

There was no statistically significant difference in 1-year FVC%pred and DLCO%pred decline between patients with high and low monocyte count [median ΔFVC%pred, derivation cohort: 0.0 (95% CI − 2.8–1.9) vs. 0.1 (95%CI − 4.0–2.2), *P* = 0.85, validation cohort: − 2.4 (95% CI − 6.3–0.7) vs. − 0.8 (95% CI − 2.0–0.4), *P* = 0.19] [median ΔDLCO%pred, derivation cohort: − 2.2 (95% CI − 4.7 to − 0.6) vs. − 2.8 (95% CI − 5.7 to − 1.2), *P* = 0.70, validation cohort: − 3.5 (95% CI − 7.1 to − 1.3) vs. − 3.3 (95% CI − 5.5 to − 1.0), *P* = 0.64] (Table 4). There was no statistically significant difference in 1-year FVC%pred and DLCO%pred decline between patients with high and low RDW [median ΔFVC%pred, derivation cohort: 0.0 (95% CI − 2.7–1.9) vs. − 2.1 (95%CI − 4.5 to − 0.1), *P* = 0.10, validation cohort: − 0.3 (95% CI-2.3–4.1) vs. − 1.9 (95% CI − 4.5 to − 0.1), *P* = 0.08] [median ΔDLCO%pred, derivation cohort: − 2.1 (95% CI − 4.5 to − 0.1) vs. − 2.1 (95% CI − 5.4 to − 1.0), *P* = 0.82, validation cohort: − 3.0 (95% CI − 6.1–1.0) vs. − 3.5 (95% CI − 5.5–1.4), *P* = 0.64] (Table 4).

**Table 4:** Median 1-year decline in FVC%pred and DLCO%pred in subgroups of patients split by median values of baseline laboratory parameters

Laboratory parameter	Parameter of functional decline	High group	Low group	*P* value
Monocyte count	Median ΔFVC%pred (derivation cohort), (95% CI)	0.0 (− 2.8 to 1.9)	0.1 (− 4.0 to 2.2)	0.85
	Median ΔFVC%pred (validation cohort), (95% CI)	− 2.4 (− 6.3 to 0.7)	− 0.8 (− 2.0 to 0.4)	0.19
	Median ΔDLCO%pred (derivation cohort), (95% CI)	− 2.2 (− 4.7 to − 0.6)	− 2.8 (− 5.7 to -1.2)	0.70
	Median ΔDLCO%pred (validation cohort), (95% CI)	− 3.5 (− 7.1 to − 1.3)	− 3.3 (− 5.5 to − 1.0)	0.64
RDW	Median ΔFVC%pred (derivation cohort), (95% CI)	0.0 (− 2.7 to 1.9)	− 2.1 (− 4.5 to − 0.1)	0.10
	Median ΔFVC%pred (validation cohort), (95% CI)	− 0.3 (− 2.3 to 4.1)	− 1.9 (− 4.5 to − 0.1)	0.08
	Median ΔDLCO%pred (derivation cohort), (95% CI)	− 2.1 (− 4.5 to − 0.1)	− 2.1 (− 5.4 to − 1.0)	0.82
	Median ΔDLCO%pred (validation cohort), (95% CI)	− 3.0 (− 6.1 to 1.0)	− 3.5 (− 5.5 to 1.4)	0.64

High and low groups indicate patients with values above and below the median of the studied parameter (monocyte count: 0.6 K/μL, RDW: 14.1%)

*ΔFVC%pred* post 1 year FVC%pred—baseline FVC%pred, *ΔDLCO%pred* post 1 year DLCO%pred- baseline DLCO%pred, *CI* confidence interval, *DLCO* diffusing capacity for carbon monoxide, *FVC* forced vital capacity, *RDW* red cell distribution width

Median monocyte count and RDW did not differ considerably between patients with 1-year ΔFVC%pred ≥ 10% and 1-year ΔFVC%pred < 10% [median monocyte count(K/μL), derivation cohort: 0.51 (95% CI 0.46–0.68) vs. 0.60 (95% CI 0.59–0.66), *P* = 0.17, validation cohort: 0.60 (95% CI 0.36–0.65) vs. 0.51 (95% CI 0.50–0.58), *P* = 0.98], [median RDW (%), derivation cohort: 14.1 (95% CI 13.5–14.7) vs. 14.2 (95% CI 14.0–14.4), *P* = 0.96, validation cohort: 13.8 (95% CI 13.0–14.0) vs. 13.7 (95% CI 13.6–13.9), *P* = 0.40] (Additional file 1: Table S2). Μedian monocyte count and RDW were not significantly different for patients with 1-year ΔDLCO%pred ≥ 15% and 1-year ΔDLCO%pred < 15% [median monocyte count(K/μL), derivation cohort: 0.59 (95% CI 0.48–0.62) vs. 0.62 (95% CI 0.59–0.68), *P* = 0.23, validation cohort: 0.45 (95% CI 0.30–0.75) vs. 0.51 (95% CI 0.50–0.60), *P* = 0.71], [median RDW (%), derivation cohort: 14.2 (95% CI 13.4–16.3) vs. 14.1 (95% CI 13.9–14.3), *P* = 0.42, validation cohort: 14.0 (95% CI 13.1–14.8) vs. 13.4 (95% CI 13.1–13.7), *P* = 0.43] (Additional file 1: Table S3).

### Effect of 1-year antifibrotic treatment

#### Effect of 1-year antifibrotic treatment on monocyte count

In descriptive analysis, median monocyte count was similar over 1-year follow-up with antifibrotic treatment with either pirfenidone [baseline vs. 1-year, derivation cohort: 0.60 (95% CI 0.54–0.65) vs. 0.58 (95% CI 0.50–0.62), *P* = 0.48, validation cohort: 0.50 (95% CI 0.47–0.58) vs. 0.55 (95%CI 0.48–0.60), *P* = 0.28, pooled analysis: 0.54 (95% CI 0.50–0.60) vs. 0.55 (95% CI 0.50–0.60), *P* = 0.77] or nintedanib [baseline vs. 1-year, derivation cohort: 0.64 (95% CI 0.50–0.70) vs. 0.61 (95% CI 0.52–0.70), *P* = 0.67, validation cohort: 0.51 (95% CI 0.50–0.59) vs. 0.50 (95%CI 0.48–0.53), *P* = 0.47, pooled analysis: 0.56 (95% CI 0.50–0.60) vs. 0.53 (95% CI 0.50–0.58), *P* = 0.49] (Additional file 1: Table S4).

#### Effect of 1-year antifibrotic treatment on RDW

In descriptive analysis, median RDW was similar over 1-year follow-up with antifibrotic treatment with either pirfenidone [baseline vs. 1-year, derivation cohort: 14.1 (95% CI 13.8–14.3) vs. 14.0 (95% CI 13.9–14.3), *P* = 0.84, validation cohort: 13.6 (95% CI 13.2–13.8) vs. 13.7 (95%CI 13.4–13.8), *P* = 0.45, pooled analysis: 13.8 (95% CI 13.5–14.0) vs. 13.8 (95% CI 13.5–13.9), *P* = 0.80] or nintedanib [baseline vs. 1-year, derivation cohort: 13.8 (95% CI 13.4–14.4) vs. 13.9 (95% CI 13.3–14.6), *P* = 0.94, validation cohort: 13.5 (95% CI 13.3–13.8) vs. 13.9 (95% CI 13.6–14.0), *P* = 0.15, pooled analysis: 13.7 (95% CI 13.4–13.8) vs. 13.9 (95% CI 13.7–14.0), *P* = 0.25] (Additional file 1: Table S5).

### All-cause mortality

#### High monocyte count correlates with increased risk of all-cause mortality

**Fig. 3:**
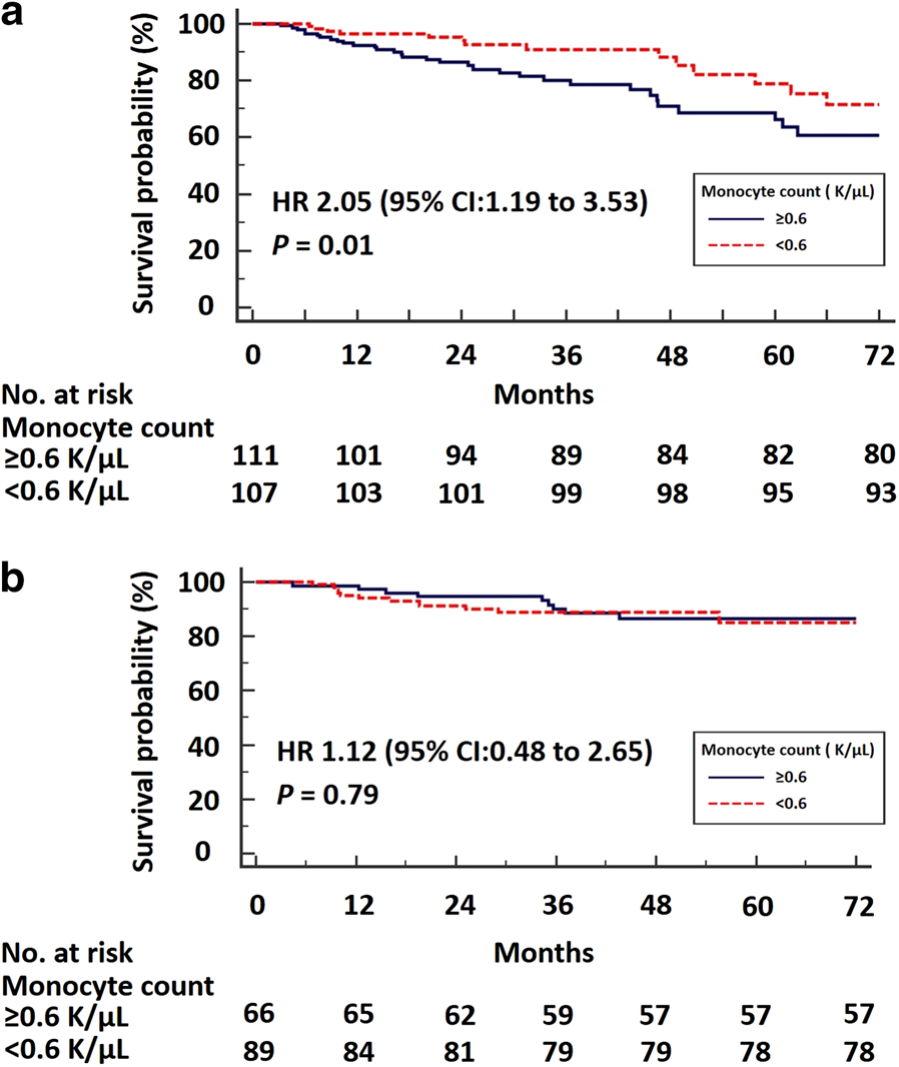
Kaplan–Meier survival curve of the derivation cohort: The cut-off threshold of 0.60 K/μL for monocyte count was used as a dichotomous variable and differentiated high from low-risk mortality groups [HR 2.05, (95% CI 1.19–3.53), (*P* = 0.01)] (**a**). Kaplan–Meier survival curve of the validation cohort: The same cut-off threshold did not reach statistical significance in the validation cohort (*P* = 0.79) (**b**)

In the derivation cohort, patients in the high monocyte group (≥ 0.60 K/μL) experienced increased risk of all-cause mortality compared to the low group (monocyte count < 0.60 K/μL) [HR 2.05, (95% CI 1.19–3.53), (*P* = 0.01)] (Fig. 3a). This finding was not confirmed in the validation cohort (*P* = 0.79) (Fig. 3b). Moreover, pooled analysis of the study population demonstrated an increased risk in all-cause mortality for patients with baseline monocyte count ≥ 0.95 K/μL compared to patients with baseline monocyte count < 0.95 Κ/μL [HR 2.47, (95% CI 0.94–6.47), (*P* = 0.005)] (Additional file 1: Figure S1). There was no increased risk of all-cause mortality for patients in the high RDW group compared to the low group, in both the derivation (*P* = 0.82) and the validation (*P* = 0.90) cohort, as well as in the pooled analysis (*P* = 0.41). Data for mortality were available for 218, 155 and 373 patients in the derivation cohort, validation cohort and pooled analysis, respectively.

## Discussion

This real-life retrospective study demonstrated that peripheral blood monocyte count was predictive of all-cause mortality in the derivation cohort and in a pooled collective of highly characterized patients with IPF. We also showed that patients with elevated levels of monocyte count and RDW exhibited more advanced disease at initial assessment compared to patients with low levels. There was no association of high monocyte count or RDW with 1-year disease progression, as assessed by functional decline. No effects of anti-fibrotic treatment on monocyte count or RDW were observed over 1-year of follow-up. Differences in baseline monocyte count, RDW, DLCO% pred and LTOT use between the two cohorts might be partially attributed to divergent endotypes across the world and/or different baseline functional status.

Our findings are consistent with those of previous reports evaluating a possible link between monocyte count and prognosis in patients with IPF [6, 25, 30, 31]. A previous retrospective, multicenter cohort study showed that monocyte count ≥ 0.95 K/μL was significantly associated with all-cause mortality compared to monocyte count < 0.95 K/μL in 7459 patients with IPF [6]. Nonetheless, IPF diagnosis in this study was based on ICD-10 medical records posing limitations to the findings. Analysis of 231 patients with IPF from the Australian registry corroborated evidence that elevated monocyte count were associated with worse clinical outcomes [30]. Most recently, pooled retrospective analysis of 2067 highly characterized patients with IPF derived from the pirfenidone trials (ASCEND, CAPACITY and INSPIRE) showed that patients with IPF and monocyte count in the range of 0.60–0.95 K/μL or ≥ 0.95 K/μL had a higher 1-year risk of IPF progression, all-cause hospitalization and all-cause mortality compared to patients with monocyte count of < 0.60 K/μL [29]. Given the results from pirfenidone clinical trials and our real-life study, monocyte count ≥ 0.60 K/μL, appears to be a highly robust and reproducible cut-off threshold which could potentially enrich the population of clinical trials, as a marker associated with greater risk of mortality and/or disease progression. In addition, it may alert clinicians in the context of risk stratification for timely interventions. In our study, monocyte count was predictive of all-cause mortality in the derivation but not the validation cohort. This might be partially attributed to the worse baseline functional status of the validation cohort, as indicated by the lower DLCO%pred and increased use of LTOT at baseline.

With regards to RDW, a previous study enrolling 319 patients with IPF reported lower median DLCO%pred and increased mortality risk for patients with RDW > 15% compared to patients with RDW ≤ 15% [25]. Our study yielded similar results for DLCO%pred. Subgroup analysis of our cohorts did not show a survival benefit for patients with RDW < 14.1%; yet, our study was designed to assess differences in subgroups based on the median RDW (14.1%) and not based on the previously published cut-off threshold of 15%.

In our study, patients with increased baseline monocyte count and RDW exhibited more advanced disease at initial assessment as indicated by baseline FVC%pred and DLCO%pred. However, monocyte count and RDW were not associated with 1-year FVC%pred and DLCO%pred decline. Similarly to our findings, recent evidence using pooled data from the TOMORROW and INPULSIS trials, showed that the adjusted rate of FVC decline was similar between patients with high and low monocyte count receiving nintedanib [32]. Nonetheless, there is still a major knowledge gap for the longitudinal prognostic and theragnostic role of these biomarkers. The prognostic role of monocyte count in FVC decline requires further investigation, as its prognostic accuracy might be associated with the baseline status, the selected treatment or the population investigated [29, 32]. Further large studies are needed to address this issue, focusing on subgroup of patients that have been subjected to different treatment modalities.

Importantly, monocyte count and RDW were similar over 1-year follow-up of antifibrotic treatment either with pirfenidone or nintedanib. RDW has been widely considered a reproducible marker, given the relatively prolonged lifespan of red blood cells [27]. Previous reports have shown that patients with a high monocyte count at diagnosis maintained their high count through the disease course [6, 17]. There was no correlation between change in monocyte count over time and survival [6]. Instead, monocyte count seemed to be relatively stable over time indicating that patients with IPF retained the same risk profile [6, 17]. To this end, monocyte count seems to have greater potential as a prognostic biomarker rather than as a predictive biomarker of treatment response; nonetheless, this requires further investigation in future cohorts applying subgroup analyses.

The prognostic role of monocyte count and RDW in patients with IPF could be partially explained from recently emerged experimental evidence suggesting migration of monocytes from the bone marrow to the injured lung and differentiation to pro-fibrotic macrophages or even fibroblasts [33–37]. Single-cell RNA sequencing characterized the heterogeneity of macrophages in bleomycin-induced pulmonary fibrosis and identified a pathological subgroup of transitional macrophages (CX3CR1 + SiglecF +) required for the fibrotic response to the injurious stimuli [38]. Recent evidence has shown that the compartmental imbalance of fractalkine mediated the migration of CX3CR1 + non-classical monocytes into fibrotic lung tissues, while non-classical monocytes-derived cells presented with a M2-like and phagocytic phenotype in fibrotic lungs [39]. Translational studies have demonstrated that accumulation of distinct populations of alveolar macrophages and higher levels of circulating fibrocytes, derived from the monocyte cell lineage, may be predictive of pulmonary fibrosis progression [36, 37, 40, 41]. Finally, C–C motif chemokine ligand 18, produced in a considerable extent by alveolar macrophages, has been suggested as a promising, serum biomarker of disease progression and mortality in patients with IPF [42].

On the other hand, a causal-effect relationship between IPF and elevated RDW is highly unlikely. Instead, it is more likely that increased RDW is indicative of patients’ hypoxemia and/or comorbidome in a similar way with other chronic lung diseases [20, 24–27]. It has been proposed that arterial hypoxemia leads to increased erythropoietin secretion and thus to increased RDW through mechanisms involving regulation of erythrocyte maturation and survival [20, 27]. Elevated RDW might have a role in the early identification of patients with IPF and intermittent hypoxemia [25]. Patients with profound hypoxemia are easily diagnosed; intermittent hypoxemia might escape routine examination and there is still a need for biomarkers contributing to their identification.

Our study has some limitations. First of all, our study has the inherent weaknesses of a retrospective study. Nonetheless, the nature of this study enabled us to report longitudinal outcomes of patients with IPF. Secondly, our sample size is moderate compared to previous reports for the prognostic role of monocyte count; yet, the size is acceptable for a real-life study. Thirdly, we had data for LTOT, but not for po2 levels; thus, we could not further investigate the association of hypoxemia with monocyte count and RDW. Moreover, data for all-cause mortality was available; yet, the specific cause of death was not available for all patients. Finally, our results should be interpreted in the context of a real-life study that may be in part influenced by other factors including steroid use prior to admission at a referral center. To this end, multiple regression analysis was performed to adjust for these covariates.

## Conclusions

This was the first real-life study of highly characterized patients with IPF showing that patients with IPF and high monocyte count (≥ 0.60 K/μL) exhibited more advanced disease at initial assessment and had a higher risk of all-cause mortality compared to patients with low monocyte count (< 0.60 K/μL). RDW failed to predict disease progression and all-cause mortality. Our study coupled with previous reports demonstrating that peripheral blood monocytes can be easily incorporated into the routine clinical assessment of patients with IPF as a reliable prognostic biomarker considering its reproducibility, cost-effectiveness and simplicity. Future prospective studies investigating the association of baseline and serial measurements of monocyte count with disease outcomes and treatment response are greatly anticipated.

## Supplementary Information

**Additional file1: **Additional Tables and Figure.

## Data Availability

The datasets used and/or analysed during the current study are available from the corresponding author on reasonable request.

## Abbreviations

CBC: Complete blood count
DLCO%pred: Diffusion capacity of lung for carbon monoxide %predicted
FVC%pred: Forced vital capacity %predicted
IPF: Idiopathic pulmonary fibrosis
LTOT: Long term oxygen therapy
RDW: Red cell distribution width

## References

[1] Raghu GR-JM, Myers JL, Richeldi L, Ryerson CJ, Lederer DJ, Behr J, Cottin V, Danoff SK, Morell F, Flaherty KR, Wells A, Martinez FJ, Azuma A, Bice TJ, Bouros D, Brown KK, Collard HR, Duggal A, Galvin L, Inoue Y, Jenkins RG, Johkoh T, Kazerooni EA, Kitaichi M, Knight SL, Mansour G, Nicholson AG, Pipavath SNJ, Buendía-Roldán I, Selman M, Travis WD, Walsh S, Wilson KC (2018). Diagnosis of idiopathic pulmonary fibrosis. An official ATS/ERS/JRS/ALAT clinical practice guideline. Am J Respir Critical Care Med.

[2] King TE, Bradford WZ, Castro-Bernardini S, Fagan EA, Glaspole I, Glassberg MK (2014). A phase 3 trial of pirfenidone in patients with idiopathic pulmonary fibrosis. N Engl J Med.

[3] Noble PW, Albera C, Bradford WZ, Costabel U, Glassberg MK, Kardatzke D (2011). Pirfenidone in patients with idiopathic pulmonary fibrosis (CAPACITY): two randomised trials. Lancet.

[4] Richeldi L, du Bois RM, Raghu G, Azuma A, Brown KK, Costabel U (2014). Efficacy and safety of nintedanib in idiopathic pulmonary fibrosis. N Engl J Med.

[5] Ley B, Collard HR, King TE (2011). Clinical course and prediction of survival in idiopathic pulmonary fibrosis. Am J Respir Crit Care Med.

[6] Scott MKD, Quinn K, Li Q, Carroll R, Warsinske H, Vallania F (2019). Increased monocyte count as a cellular biomarker for poor outcomes in fibrotic diseases: a retrospective, multicentre cohort study. Lancet Respir Med.

[7] Neighbors M, Cabanski CR, Ramalingam TR, Sheng XR, Tew GW, Gu C (2018). Prognostic and predictive biomarkers for patients with idiopathic pulmonary fibrosis treated with pirfenidone: post-hoc assessment of the CAPACITY and ASCEND trials. Lancet Respir Med.

[8] Tzouvelekis A, Herazo-Maya JD, Slade M, Chu JH, Deiuliis G, Ryu C (2017). Validation of the prognostic value of MMP-7 in idiopathic pulmonary fibrosis. Respirology.

[9] Maher TM, Oballa E, Simpson JK, Porte J, Habgood A, Fahy WA (2017). An epithelial biomarker signature for idiopathic pulmonary fibrosis: an analysis from the multicentre PROFILE cohort study. Lancet Respir Med.

[10] Jenkins RG, Simpson JK, Saini G, Bentley JH, Russell AM, Braybrooke R (2015). Longitudinal change in collagen degradation biomarkers in idiopathic pulmonary fibrosis: an analysis from the prospective, multicentre PROFILE study. Lancet Respir Med.

[11] Raghu G, Richeldi L, Jagerschmidt A, Martin V, Subramaniam A, Ozoux ML (2018). Idiopathic pulmonary fibrosis: prospective, case-controlled study of natural history and circulating biomarkers. Chest.

[12] Stuart BD, Lee JS, Kozlitina J, Noth I, Devine MS, Glazer CS (2014). Effect of telomere length on survival in patients with idiopathic pulmonary fibrosis: an observational cohort study with independent validation. Lancet Respir Med.

[13] Richards TJ, Kaminski N, Baribaud F, Flavin S, Brodmerkel C, Horowitz D (2012). Peripheral blood proteins predict mortality in idiopathic pulmonary fibrosis. Am J Respir Crit Care Med.

[14] Organ LA, Duggan AR, Oballa E, Taggart SC, Simpson JK, Kang'ombe AR (2019). Biomarkers of collagen synthesis predict progression in the PROFILE idiopathic pulmonary fibrosis cohort. Respir Res.

[15] Peljto AL, Zhang Y, Fingerlin TE, Ma SF, Garcia JG, Richards TJ (2013). Association between the MUC5B promoter polymorphism and survival in patients with idiopathic pulmonary fibrosis. JAMA.

[16] Oldham JM, Ma SF, Martinez FJ, Anstrom KJ, Raghu G, Schwartz DA (2015). TOLLIP, MUC5B, and the response to N-acetylcysteine among individuals with idiopathic pulmonary fibrosis. Am J Respir Crit Care Med.

[17] Herazo-Maya JD, Sun J, Molyneaux PL, Li Q, Villalba JA, Tzouvelekis A (2017). Validation of a 52-gene risk profile for outcome prediction in patients with idiopathic pulmonary fibrosis: an international, multicentre, cohort study. Lancet Respir Med.

[18] Tzouvelekis A, Herazo-Maya J, Sakamoto K, Bouros D (2016). Biomarkers in the evaluation and management of idiopathic pulmonary fibrosis. Curr Top Med Chem.

[19] Kreuter M, Maher TM (2019). Can monocytes predict prognosis of idiopathic pulmonary fibrosis?. Lancet Respir Med.

[20] Karampitsakos T, Dimakou K, Papaioannou O, Chrysikos S, Kaponi M, Bouros D, et al. The role of increased red cell distribution width as a negative prognostic marker in patients with COPD. Pulmonary Pharmacol Therapeutics. 2019:101877. 10.1016/j.pupt.2019.101877 31843703

[21] Suissa S, Dell'Aniello S, Ernst P (2018). Comparative effectiveness of LABA-ICS versus LAMA as initial treatment in COPD targeted by blood eosinophils: a population-based cohort study. Lancet Respir Med.

[22] Doroudchi A, Pathria M, Modena BD (2020). Asthma biologics: comparing trial designs, patient cohorts and study results. Ann Allergy, Asthma Immunol: Off Publ Am College Allergy, Asthma, Immunol.

[23] Teoh AKY, Jo HE, Chambers DC, Symons K, Walters EH, Goh NS, et al. Blood monocyte counts as a potential prognostic marker for idiopathic pulmonary fibrosis: analysis from the Australian IPF registry. Eur Respir J. 2020;55(4). 10.1183/13993003.01855-2019 31949112

[24] Hampole CV, Mehrotra AK, Thenappan T, Gomberg-Maitland M, Shah SJ (2009). Usefulness of red cell distribution width as a prognostic marker in pulmonary hypertension. Am J Cardiol.

[25] Nathan SD, Reffett T, Brown AW, Fischer CP, Shlobin OA, Ahmad S (2013). The red cell distribution width as a prognostic indicator in idiopathic pulmonary fibrosis. Chest.

[26] Braun E, Domany E, Kenig Y, Mazor Y, Makhoul BF, Azzam ZS (2011). Elevated red cell distribution width predicts poor outcome in young patients with community acquired pneumonia. Critical Care (London, England).

[27] Epstein D, Nasser R, Mashiach T, Azzam ZS, Berger G (2018). Increased red cell distribution width: a novel predictor of adverse outcome in patients hospitalized due to acute exacerbation of chronic obstructive pulmonary disease. Respir Med.

[28] Karampitsakos T, Akinosoglou K, Papaioannou O, Panou V, Koromilias A, Bakakos P (2020). Increased red cell distribution width is associated with disease severity in hospitalized adults with SARS-CoV-2 infection: an observational multicentric study. Front Med.

[29] Kreuter M, Bradley SJ, Lee JS, Tzouvelekis A, Oldham JM, Molyneaux PL, et al. Monocyte count as a prognostic biomarker in patients with idiopathic pulmonary fibrosis. Am J Respir Crit Care Med 2021. 10.1164/rccm.202003-0669OC PMC8437112 33434107

[30] Teoh AKY, Jo HE, Chambers DC, Symons K, Walters EH, Goh NS, et al. Blood monocyte counts as a potential prognostic marker for IPF: analysis from the Australian IPF registry. Eur Respir J. 2020;1901855. 10.1183/13993003.01855-2019 31949112

[31] Kawamura K, Ichikado K, Anan K, Yasuda Y, Sekido Y, Suga M (2020). Monocyte count and the risk for acute exacerbation of fibrosing interstitial lung disease: a retrospective cohort study. Chron Respir Dis.

[32] Tzouvelekis A, Maher TM, Goh N, Kreuter M, Cottin V, Schinzel B (2020). Monocyte count and decline in forced vital capacity (FVC) in patients with IPF. Eur Respir J.

[33] Lederer DJ, Martinez FJ (2018). Idiopathic pulmonary fibrosis. N Engl J Med.

[34] Betensley A, Sharif R, Karamichos D (2016). A systematic review of the role of dysfunctional wound healing in the pathogenesis and treatment of idiopathic pulmonary fibrosis. J Clin Med.

[35] Yu G, Tzouvelekis A, Wang R, Herazo-Maya JD, Ibarra GH, Srivastava A (2018). Thyroid hormone inhibits lung fibrosis in mice by improving epithelial mitochondrial function. Nat Med.

[36] Allden SJ, Ogger PP, Ghai P, McErlean P, Hewitt R, Toshner R (2019). The transferrin receptor CD71 delineates functionally distinct airway macrophage subsets during idiopathic pulmonary fibrosis. Am J Respir Crit Care Med.

[37] Heukels P, van Hulst JAC, van Nimwegen M, Boorsma CE, Melgert BN, van den Toorn LM (2018). Fibrocytes are increased in lung and peripheral blood of patients with idiopathic pulmonary fibrosis. Respir Res.

[38] Aran D, Looney AP, Liu L, Wu E, Fong V, Hsu A (2019). Reference-based analysis of lung single-cell sequencing reveals a transitional profibrotic macrophage. Nature Immunol.

[39] Greiffo FR, Viteri-Alvarez V, Frankenberger M, Dietel D, Ortega-Gomez A, Lee JS, et al. CX3CR1-fractalkine axis drives kinetic changes of monocytes in fibrotic interstitial lung diseases. 2020;55(2). 10.1183/13993003.00460-2019 31744836

[40] Nouno T, Okamoto M, Ohnishi K, Kaieda S, Tominaga M, Zaizen Y (2019). Elevation of pulmonary CD163(+) and CD204(+) macrophages is associated with the clinical course of idiopathic pulmonary fibrosis patients. J Thorac Dis.

[41] Moeller A, Gilpin SE, Ask K, Cox G, Cook D, Gauldie J (2009). Circulating fibrocytes are an indicator of poor prognosis in idiopathic pulmonary fibrosis. Am J Respir Crit Care Med.

[42] Prasse A, Probst C, Bargagli E, Zissel G, Toews GB, Flaherty KR (2009). Serum CC-chemokine ligand 18 concentration predicts outcome in idiopathic pulmonary fibrosis. Am J Respir Crit Care Med.

