# Supplementary material for: Increased monocyte count and red cell distribution width as prognostic biomarkers in patients with Idiopathic Pulmonary Fibrosis
Source: Respir Res. 2021 May 5;22:140. doi: 10.1186/s12931-021-01725-9 (PMC8097815; doi:10.1186/s12931-021-01725-9)

**Supplementary data**

**Increased monocyte count and red cell distribution width as prognostic biomarkers in patients with Idiopathic Pulmonary Fibrosis.**

Theodoros Karampitsakos^1^, Sebastiano Torrisi^2^, Katerina Antoniou^3^, Effrosyni Manali^4^, Ioanna Korbila^4^, Ourania Papaioannou^1^, Fotios Sampsonas^1^, Matthaios Katsaras^1^, Eirini Vasarmidi ^3^, Despoina Papakosta^5^, Kalliopi Domvri^5^, Eva Fouka^5^, Ioannis Organtzis^5^, Zoe Daniil^6^, Ilias Dimeas^6^, Paraskevi Kirgou^6^, Konstantinos I Gourgoulianis^6^, Ilias C Papanikolaou^7^, Katerina Markopoulou^8^, Georgia Kounti^8^, Eirini Tsapakidou^8^, Efthymia Papadopoulou^8^, Konstantinos Tatsis^9^, Athena Gogali^9^, Konstantinos Kostikas^9^, Vasilios Tzilas^10^, Serafeim Chrysikos^11^, Spyridon Papiris^4^, Demosthenes Bouros^10*^, Michael Kreuter^2*^, Argyrios Tzouvelekis ^1,10*^

**Equally supervised submitted work*

^1^ Department of Respiratory Medicine, University Hospital of Patras, Patras, Greece

^2^ Center for interstitial and rare lung diseases, Pneumology, Thoraxklinik, University of Heidelberg, Germany and German Center for Lung Research, Heidelberg, Germany

^3^ Laboratory of Molecular and Cellular Pneumonology, Department of Respiratory Medicine, Faculty of

Medicine, University of Crete, Heraklion, Crete, Greece

^4^ 2^nd^ Pulmonary Medicine Department, "ATTIKON" University Hospital, Athens Medical School, National and Kapodistrian University of Athens, Athens, Greece

^5^Pulmonary Department, Medical School, Aristotle University of Thessaloniki, ‘’G. PAPANIKOLAOU’’ General Hospital, Exochi, Thessaloniki, Greece

^6^ Department of Respiratory Medicine, Medical School, University of Thessaly, Larissa, Greece

^7^ Respiratory Medicine Department, "Corfu General Hospital", Corfu, Greece

^8^ Pulmonary Department “G. PAPANIKOLAOU” General Hospital, Thessaloniki, Greece

^9^ Department of Respiratory Medicine, Medical School, University of Ioannina, Ioannina, Greece

^10^ First Academic Department of Pneumonology, Hospital for Thoracic Diseases, “SOTIRIA”, Medical School, National and Kapodistrian University of Athens, Athens, Greece

^11^ 5^th^ Department of Pneumonology, Hospital for Thoracic Diseases, “SOTIRIA”, Athens, Greece

*Correspondence to:*

Argyrios Tzouvelekis MD, MSc, PhD

Associate Professor of Respiratory Medicine

Head Department of Respiratory Medicine

University of Patras, Greece

[*atzouvelekis@upatras.gr*](mailto:atzouvelekis@upatras.gr)*,* [*argyrios.tzouvelekis@fleming.gr*](mailto:argyrios.tzouvelekis@fleming.gr)

**Supplemental Table 1. Multiple regression analysis showing the impact of comorbidities on baseline monocyte count and RDW.**

|  | **Monocyte count** | | |  |  | | |  | | | **RDW** | | | |  | | |  |  |  |
| --- | --- | --- | --- | --- | --- | --- | --- | --- | --- | --- | --- | --- | --- | --- | --- | --- | --- | --- | --- | --- |
| **Parameter** | | **Coefficient** | **Std**  **Error** | | | | **p**  **value** | | | **Coefficient** | | | **Std**  **Error** | | | | ***P***  **value** | | |  |
|  | |  |  | | |  | | |  | | | | |  | |  |  |  |  |  |
| Age  Gender  Current smoker  Ever smoker  Never smoker  AH  PH  GERD  DM  Thyroid disorders | | 0.002191  -0.07621  -0.01933  -0.1135  -0.1117  -0.01524  0.05899  -0.002937  -0.01533  -0.05273 | | 0.001332  0.02821  0.1128  0.1053  0.1068  0.02187  0.03010  0.02377  0.02495  0.03600 | | | 0.10  **0.007**  0.86  0.28  0.30  0.49  0.05  0.90  0.54  0.14 | | | 0.008626  0.1649  0.9684  0.7481  0.7960  0.2913  0.8759  -0.01597  0.1174  -0.05379 | | 0.01016  0.2165  0.8548  0.7937  0.8057  0.1688  0.2329  0.1835  0.1915  0.2782 | | | | | | | 0.40  0.45  0.26  0.35  0.32  0.09  **0.0002**  0.93  0.54  0.85 | |

**Abbreviations:** AH: arterial hypertension, DM: diabetes mellitus, GERD: gastroesophageal reflux disease, Hb: hemoglobin, PH: pulmonary hypertension, RDW: red cell distribution width, Std: Standard.

**Supplemental Table 2. Monocyte count** (**K/μL) and RDW (%) in subgroup of patients based on post 1-year FVC decline.**

|  | **ΔFVC%pred≥10%** | | | **ΔFVC%pred<10%** | ***P***  **value** | |
| --- | --- | --- | --- | --- | --- | --- |
|  |  | | |  |  | |
| Median Monocyte count (derivation cohort), (95%CI)  Median Monocyte count (validation cohort), (95%CI)  Median RDW (derivation cohort), (95%CI)  Median RDW (validation cohort), (95%CI) | 0.51 (0.46 to 0.68)  0.60 (0.36 to 0.65)  14.1 (13.5 to 14.7)  13.8 ( 13.0 to 14.0) | | | 0.60 (0.59 to 0.66)  0.51 (0.50 to 0.58)  14.2 ( 14.0 to 14.4)  13.7 (13.6 to 13.9) | 0.17  0.98  0.96  0.40 | |
|  |  |  |  | | |  |

**Abbreviations:** ΔFVC%pred: (post 1 year FVC%pred – baseline FVC%pred), CI: confidence interval, FVC: forced vital capacity, RDW: red cell distribution width.

**Supplemental Table 3. Monocyte count (K/μL) and RDW (%) in subgroup of patients based on post 1-year DLCO decline.**

|  | **ΔDLCO%pred≥15%** | | | **ΔDLCO%pred<15%** | ***P***  **value** | |
| --- | --- | --- | --- | --- | --- | --- |
|  |  | | |  |  | |
| Median Monocyte count (derivation cohort), (95%CI)  Median Monocyte count (validation cohort), (95%CI)  Median RDW (derivation cohort), (95%CI)  Median RDW (validation cohort), (95%CI) | 0.59 (0.48 to 0.62)  0.45 (0.30 to 0.75)  14.2 (13.4 to 16.3)  14.0 (13.1 to 14.8) | | | 0.62 (0.59 to 0.68)  0.51 (0.50 to 0.60)  14.1 (13.9 to 14.3)  13.4 ( 13.1 to 13.7) | 0.23  0.71  0.42  0.43 | |
|  |  |  |  | | |  |

**Abbreviations:** ΔDLCO%pred: (post 1 year DLCO%pred- baseline DLCO%pred), CI: confidence interval, DLCO: diffusing capacity for carbon monoxide, RDW: red cell distribution width.

**Supplemental Table 4. Effect of 1-year antifibrotic treatment on monocyte count (K/μL).**

|  | **Median monocyte count (95% CI)**  **Treatment--naïve** | | | **Median monocyte count (95% CI)**  **1-year post antifibrotics** | ***P***  **value** | |
| --- | --- | --- | --- | --- | --- | --- |
|  |  | | |  |  | |
| Pirfenidone (derivation cohort)  Pirfenidone (validation cohort)  Pirfenidone (pooled analysis)  Nintedanib (derivation cohort)  Nintedanib (validation cohort)  Nintedanib ( pooled analysis) | 0.60 (0.54 to 0.65)  0.50 (0.47 to 0.58 )  0.54 (0.50 to 0.60)  0.64 ( 0.50 to 0.70)  0.51 (0.50 to 0.59)  0.56 (0.50 to 0.60) | | | 0.58 (0.50 to 0.62)  0.55 ( 0.48 to 0.60)  0.55 (0.50 to 0.60)  0.61 (0.52 to 0.70)  0.50 (0.48 to 0.53)  0.53 (0.50 to 0.58) | 0.48  0.28  0.77  0.67  0.47  0.49 | |
|  |  |  |  | | |  |

**Abbreviations:** CI: confidence interval

**Supplemental Table 5. Effect of 1-year antifibrotic treatment on RDW (%).**

|  | **Median RDW**  **(95% CI)**  **Treatment--naïve** | | | **Median RDW**  **(95% CI)**  **1-year post antifibrotics** | ***P***  **value** | |
| --- | --- | --- | --- | --- | --- | --- |
|  |  | | |  |  | |
| Pirfenidone (derivation cohort)  Pirfenidone (validation cohort)  Pirfenidone (pooled analysis)  Nintedanib (derivation cohort)  Nintedanib (validation cohort)  Nintedanib ( pooled analysis) | 14.1 (13.8 to 14.3)  13.6 (13.2 to 13.8)  13.8 (13.5 to 14.0)  13.8 (13.4 to 14.4)  13.5 (13.3 to 13.8)  13.7 (13.4 to 13.8) | | | 14.0 (13.9 to 14.3)  13.7 (13.4 to 13.8)  13.8 (13.5 to 13.9)  13.9 (13.3 to 14.6)  13.9 (13.6 to 14.0)  13.9 (13.7 to 14.0) | 0.84  0.45  0.80  0.94  0.15  0.25 | |
|  |  |  |  | | |  |

**Abbreviations:** CI: confidence interval

**Supplemental Figure 1.** Kaplan-Meier survival curve of the pooled analysis: Pooled analysis of the study population demonstrated an increased risk in all-cause mortality for patients with baseline monocyte count≥ 0.95 K/μL compared to patients with baseline monocyte count <0.95 Κ/μL [HR: 2.47, (95% CI: 0.94 to 6.47), (*P*=0.005)].


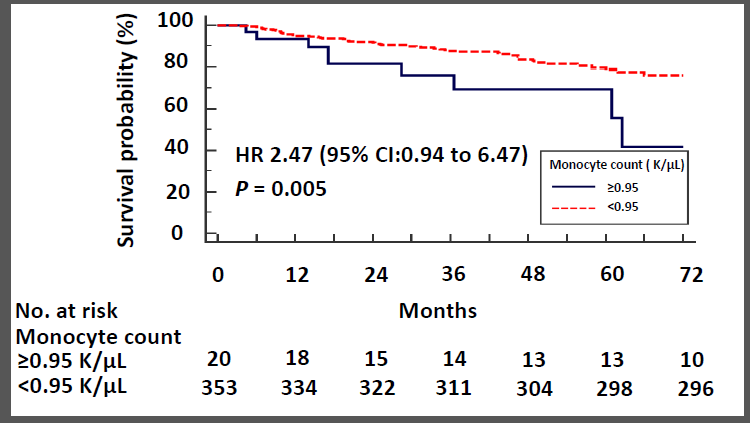

Supplement: Supplementary file 1 — Additional file1: Additional Tables and Figure. [file 12931_2021_1725_MOESM1_ESM.docx]
